# Effect of SNPs on Litter Size in Swine

**DOI:** 10.3390/cimb46070378

**Published:** 2024-06-24

**Authors:** Zhenhua Guo, Lei Lv, Di Liu, Hong Ma, Čedomir Radović

**Affiliations:** 1 Key Laboratory of Combining Farming and Animal Husbandry, Ministry of Agriculture and Rural Affairs, Institute of Animal Husbandry, Heilongjiang Academy of Agricultural Sciences, No. 368 Xuefu Road, Harbin 150086, China; 2 Wood Science Research Institute, Heilongjiang Academy of Forestry, No. 134 Haping Road, Harbin 150080, China; 3 Department of Pig Breeding and Genetics, Institute for Animal Husbandry, Autoput 16, 11080 Belgrade, Serbia

**Keywords:** gene set enrichment analysis, litter size, network meta-analysis, single-nucleotide polymorphism, PCR-RFLP/SSCP

## Abstract

Although sows do not directly enter the market, they play an important role in piglet breeding on farms. They consume large amounts of feed, resulting in a significant environmental burden. Pig farms can increase their income and reduce environmental pollution by increasing the litter size (LS) of swine. PCR-RFLP/SSCP and GWAS are common methods to evaluate single-nucleotide polymorphisms (SNPs) in candidate genes. We conducted a systematic meta-analysis of the effect of SNPs on pig LS. We collected and analysed data published over the past 30 years using traditional and network meta-analyses. Trial sequential analysis (TSA) was used to analyse population data. Gene set enrichment analysis and protein–protein interaction network analysis were used to analyse the GWAS dataset. The results showed that the candidate genes were positively correlated with LS, and defects in PCR-RFLP/SSCP affected the reliability of candidate gene results. However, the genotypes with high and low LSs did not have a significant advantage. Current breeding and management practices for sows should consider increasing the LS while reducing lactation length and minimizing the sows’ non-pregnancy period as much as possible.

## 1. Introduction

On March 2023, the number of pigs worldwide stood at 784 million (www.statista.com, accessed on 17 July 2023). In December 2022, the number of sows worldwide was 76 million. Sows do not directly enter the market but are raised on farms to breed piglets. These sows consume large amounts of feed and induce a significant environmental burden. Based on an estimated 10 litter size (LS) and two litters per year, an increase of 0.1 LS can reduce the breeding of approximately 760,000 sows. Therefore, many studies have focused on improving LS. These methods include, but are not limited to, the PCR-RFLP/SSCP verification of candidate genes [1], genome-wide association studies (GWASs) [2], QTL [3], epigenetics [4], mitochondrial DNA [5], micro RNA (miRNA) [6,7], long noncoding RNAs (lncRNAs) [8], and circular RNAs (circRNAs) [9]. The studies mentioned above were conducted based on the genetic perspective. In addition, diseases [10], feeding management [11], the order of parity [12], metabolite analysis [13], gut microbiota [14], and endocrine-disrupting chemicals (EDCs) [15] can all affect pig LS.

Single-nucleotide polymorphisms (SNPs) can affect LS in pigs, and polymerase chain reaction–restricted fragment length polymorphism (PCR-RFLP) and polymerase chain reaction–single-strand conformational polymorphism (PCR-SSCP) are traditional methods for screening SNPs. It is generally accepted that SNPs in the same gene have a consistent impact on pig LS [16]. There are also reports that the *PRLR* SNP of Large White (LW) pigs significantly affects LS but does not affect the LS of the Danish Landrace [17]. However, in *PRLR* SNP studies targeting different lines of LW pigs, lines A and B were treated with *Alu I* and achieved opposite results [18]. Hebao pig’s BB is lower than AA, while Landrace’s BB is higher than AA [19]. The *PRLR* effect on Polish pigs’ second and third parity LS exhibited the opposite results [20]. LS is regulated by several genes. Some reports have suggested the involvement of multiple genes. The same pig population can be divided into groups based on SNPs in different genes [21]. Thus, multiple SNPs in genes were merged to evaluate their effects on LS [22].

With the development of high-throughput sequencing technology, reports on the impact of SNP analysis on pig LS using GWASs have gradually increased, with related reports on nearly 2000 SNPs. However, the repeatability of different reported studies is poor, and GWAS results for the same breed differ [23]. Therefore, it is necessary to conduct a systematic meta-analysis of the effects of SNPs on pig LS. This study aimed to collect and integrate the association between pig SNPs and LS, combined with the impact of candidate gene SNPs in a meta-analysis, to provide suggestions for the future breeding of high-LS pig breeds.

## 2. Materials and Methods

This study was not pre-registered on PROSPERO/INPLASY.

### 2.1. Database Search Strategy and Study Inclusion

Two independent authors conducted the search separately. The search strategy for SNPs used the following keywords: (pig OR porcine OR swine OR hog OR boar OR sow OR piglet) AND (litter size) OR (number born) OR (number pigs farrowed) AND polymorphism. The period was from 1 January 1993 to 1 March 2023. The retrieval results discussed in this section were used to compare and analyse the effect of SNPs on pig LS. Searches for GWASs were also carried out using the same method, replacing the keyword “polymorphism” with “GWAS”. The results of this search were used to compare and analyse the SNP effects on pig LS for a specific gene. The retrieved databases included Scopus, PubMed, and the Web of Science. The inclusion criteria were as follows: the study focused on swine, the manuscript was written in English, the study contained LS data with standard deviation (SD) or standard error (SE), and GWASs listed the candidate SNPs for LW. The exclusion criteria for papers were as follows: there were no data about pigs, the manuscript was not written in English, and neither the SD or SE for LS were included.

### 2.2. Data Extraction

Due to the lack of specific details in some studies regarding gel electrophoresis and the inability to distinguish specific genotypes, piglet data were defined as either having a low LS or a high LS. Only the highest and lowest values were extracted if three genotypes were encountered. This study aimed to determine whether target gene SNPs affected LS. Therefore, the actual extracted data were the absolute values of the LS data. Specifically, in the study of SNPs, LS, newborn litter weight, weaning number, and weaning litter weight were extracted, as were the total number of populations, number of low-LS individuals, and number of high-LS individuals. Each treatment served as a dataset, creating a new dataset, A. A dataset consisting of short interspersed nuclear elements (SINEs) was used as an SNP dataset for analysis [24]. The genes summarized from the research results in the GWASs were combined to create a new dataset, B. If the same gene was mentioned for multiple SNPs, it was treated as a single gene.

### 2.3. Traditional and Network Meta-Analysis

If there were more than seven reports on the same gene, and data could be extracted from all seven reports, a traditional meta-analysis was conducted on this gene. The review manager (version 5.4) was used to execute the conversion of SE in dataset A to SD. A random model was used if the heterogeneity was greater than 50%. If the method of combining varieties and parity could be used to reduce heterogeneity to less than 50%, subgroup analysis was used. However, this resulted in too many subgroups; therefore, a random model analysis was used. Stata (version 15.0) executed the Egger and Begg test for publication bias.

The distribution pattern of genotypes in the swine population was studied, and the percentage of low and high-LS genotypes in the population was analysed. One-way analysis of variance (ANOVA) was performed using SPSS (version 25.0). The calculated results were plotted using GraphPad Prism (version 9.0.2). To clarify the genotype changes over time in the population, trial sequential analysis (TSA) was performed on the genotype data of low and high LSs using Java (version 1.8.0).

The dataset of the traditional meta-analysis was selected to simultaneously study two or more gene reports in one study for network meta-analysis. The value used was the percentage increase in LS, and the formula was as follows:

(1) $$LS\;increase\;rate=\frac{High\;LS-Low\;LS}{Low\;LS}\times 100\%$$

Network meta-analysis was performed using R software (version 4.1.2) and JAGS (version 4.3.0) with “coda”, “rjag”, and “gemtc” packages. Network image processing was performed using Gephi (version 0.10.1) for rendering.

### 2.4. Gene Set Enrichment Analysis and PPI Network Analysis

Candidate genes for dataset B, DAVID (https://david.ncifcrf.gov, accessed on 7 May 2023), were used to complete the GO and KEGG enrichment analyses. Considering the enrichment of immune-related genes, the QTL database (www.animalgenome.org, accessed on 7 May 2023), containing genes related to LS, was subjected to GO and KEGG enrichment analyses. The visualisation of the results was completed using GraphPad Prism (version 9.0.2).

Merging candidate genes from dataset B and QTL databases for PPI network construction analysis, STRING (https://cn.string-db.org/, accessed on 7 May 2023) was used to establish a PPI network. The visualisation of the results was completed using Cytoscape (version 3.7.2).

### 2.5. Homology Modelling of ESR 3D Structures and E_2_ Docking

According to the *ESR* SNP 388G>A mentioned in the literature [21], NCBI data (gene ID: AY357117) were downloaded. AY357117 is an ESR fragment containing 521 bases located at SNP 388G>A. The pig *ESR* (gene ID: 396697) was then compared in the NCBI to locate this SNP site at the full length of the gene. Translated to obtain the amino acid sequences, this SNP resulted in a 317V>M mutation. A homology model was created using amino acid sequences from the Swiss Model (https://swissmodel.expasy.org/, accessed on 27 May 2023). As ESR is a classic intracellular receptor in the nucleus and does not have a transmembrane structure, it does not simulate the transmembrane structure of proteins.

The structure of oestrogen (E_2_) was downloaded from PubChem. The SDF format files were converted to PDB format files using OpenBabel. AutoDock (version 4.2.6) conducts protein small macromolecular docking and calculates the affinity. The visualisation of the results was completed using PyMOL (version 2.6.0).

## 3. Results

### 3.1. Traditional Meta-Analysis

**Figure 1:**
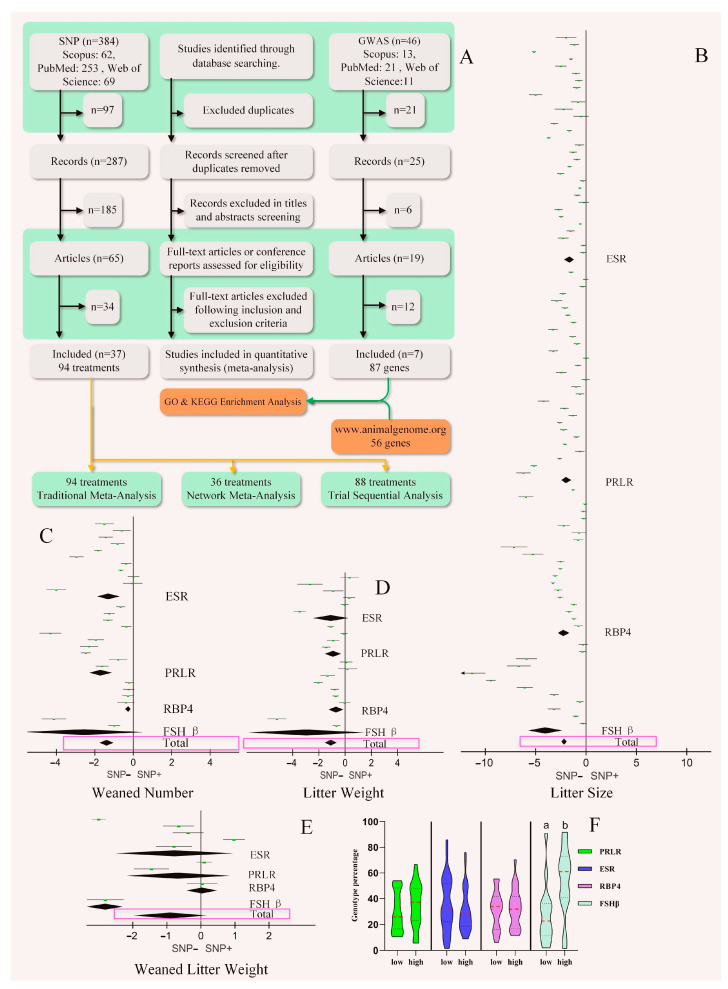
PRISMA diagram of the study selection process and traditional meta-analysis. (**A**) The time range was the past 30 years (1993–2023). Three databases were searched: PubMed, Web of Science, and Scopus. A total of 384 relevant studies on single-nucleotide polymorphisms (SNPs) and 46 on genome-wide association studies were identified. Thirty-seven studies (and 94 datasets) were selected for inclusion in this meta-analysis study. In addition, seven studies (87 + 56 genes) were selected for Gene Ontology and Kyoto Encyclopaedia of Genes and Genomes enrichment analysis. (**B**) Forest plot of *ESR*, *PRLR*, *RBP4*, and *FSH β* SNPs’ effects on swine litter size (LS). (**C**–**E**) Forest plot showing the effects of SNPs in four genes on swine weaned number, litter weight, and weaned litter weight. The black diamond block represents 95% CI. (**F**) The distribution of violin plot of four gene SNPs in the population. Different letters indicate significant differences (*p* < 0.05).

Over the past 30 years, a total of 114 papers have been published on the effect of SNPs on LS (Supplementary Table S1). After screening, the genes on which there were more than seven studies were *ESR*, *PRLR*, *RBP4*, and *FSH β*. A total of 94 datasets from 37 papers were used for LS traditional meta-analysis [1,12,16,17,18,19,20,21,22,24,25,26,27,28,29,30,31,32,33,34,35,36,37,38,39,40,41,42,43,44,45,46,47,48,49,50,51], as shown in Figure 1A.

Figure 1B shows that *ESR*, *PRLR*, *RBP4*, and *FSH β* SNPs were positively correlated with the absolute value of LS. Egger (*p* = 0.568) and Begg (Pr > |z| = 0.986) tests found no publication bias. These four genes were positively correlated with the weaned number and litter weight (Figure 1C,D). However, Figure 1E shows that these genes did not correlate with the weaned litter weight. The distribution pattern of genotypes in the population is shown in Figure 1F, and the genotypes *ESR*, *PRLR*, and *RBP4* were compared according to high and low LSs, with no significant difference. Only the *FSH β* SNPs genotype showed significant differences in the population (*p* = 0.012), with the high-LS genotype being dominant. It is worth noting that if the data for the precise positioning of SNPs were deleted [24], the *FSH β* genotype SNPs in the population were not significantly different (*p* = 0.054).

### 3.2. Network Meta-Analysis and Trial Sequential Analysis

**Figure 2:**
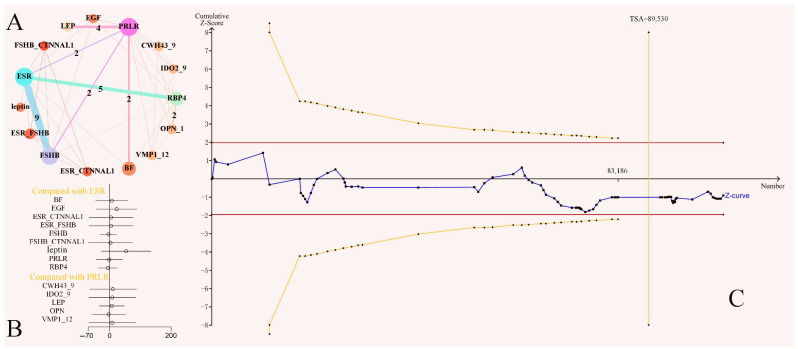
Network meta-analysis and trial sequential analysis (TSA). (**A**) Network plot showing the effects of single-nucleotide polymorphisms (SNPs) on swine LS. The circular area represents the number of sows studied, and the straight line represents the number of datasets. (**B**) Forest plot of the comparison among multiple genes; the 95% CI threshold contains 0, indicating that there was no difference in the direct evidence comparison results. (**C**) TSA of low- and high-LS SNP genotype distribution in the population. The red line represents the traditional significance horizontal line. The orange line represents the TSA threshold, and the TSA mathematical expected value is 89,530.

A network meta-analysis was performed on 36 datasets, and Figure 2A shows the network plot, where the two-arm, three-arm, and five-arm datasets were 19, 2, and 3, respectively. Figure 2B shows the LS effect compared to multiple genes. There were no differences among the 15 candidate genes. Figure 2C shows the TSA results; the accumulated information exceeded the expected confidence value of 89,530. However, there was no statistically significant difference between the low- and high-LS genotypes, indicating that there was no significant difference in the distribution of the low and high-LS genotypes in the population.

### 3.3. Gene Enrichment Analysis and Protein–Protein Interaction (PPI) Network Analysis

**Figure 3:**
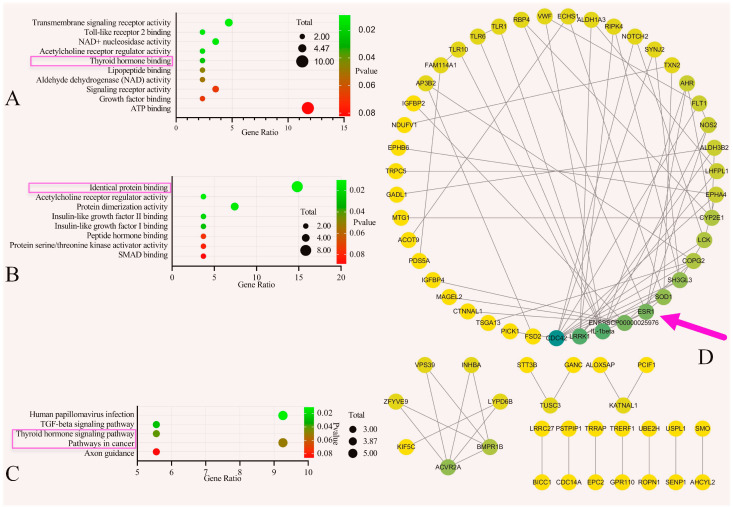
Gene enrichment and protein–protein interaction network. (**A**) Bubble plot of the 87 genes from the Gene Ontology–MF results collected from genome-wide association studies on SNPs’ effect on swine litter size (LS). (**B**) Bubble plot of the 56 genes from the GO-MF results downloaded from QTL data (www.animalgenome.org) about LS. (**C**) Bubble plot of the 56 genes from the Kyoto Encyclopedia of Genes and Genomes results from the QTL data. (**D**) Protein–protein interaction network merging the 87 and 56 genes. The pink arrow indicates the *ESR* gene.

SNPs affect the LS of pigs, and 87 genes were collected from seven articles regarding the inclusion of GWASs, which are listed in Table 1. The QTL database (www.animalgenome.org) downloaded 56 genes. The results of the GO enrichment analysis are shown in Figure 3A,B. The top four genes in the 87 genes from the GO-MF results were all immune-related pathways. In fifth place was the thyroid hormone binding protein. The GO-MF results for the 56 genes were identical to those for protein binding. The labelled genes contained *ESR*. Figure 3C shows the KEGG enrichment analysis results for the 56 genes with thyroid hormone signalling pathways and pathways in cancer. The labelled genes also contained an *ESR* signal. After merging 87 and 56 genes and removing duplicate genes, the protein–protein interaction (PPI) network analysis results for 126 genes were obtained, as shown in Figure 3D. The pink arrow indicates that the *ESR* gene is arranged in fifth place. Therefore, we conducted homology modelling and E_2_ docking on the SNP of the *ESR* gene.

### 3.4. Homology Modelling of ESR 3D Structures and E_2_ Docking

**Figure 4:**
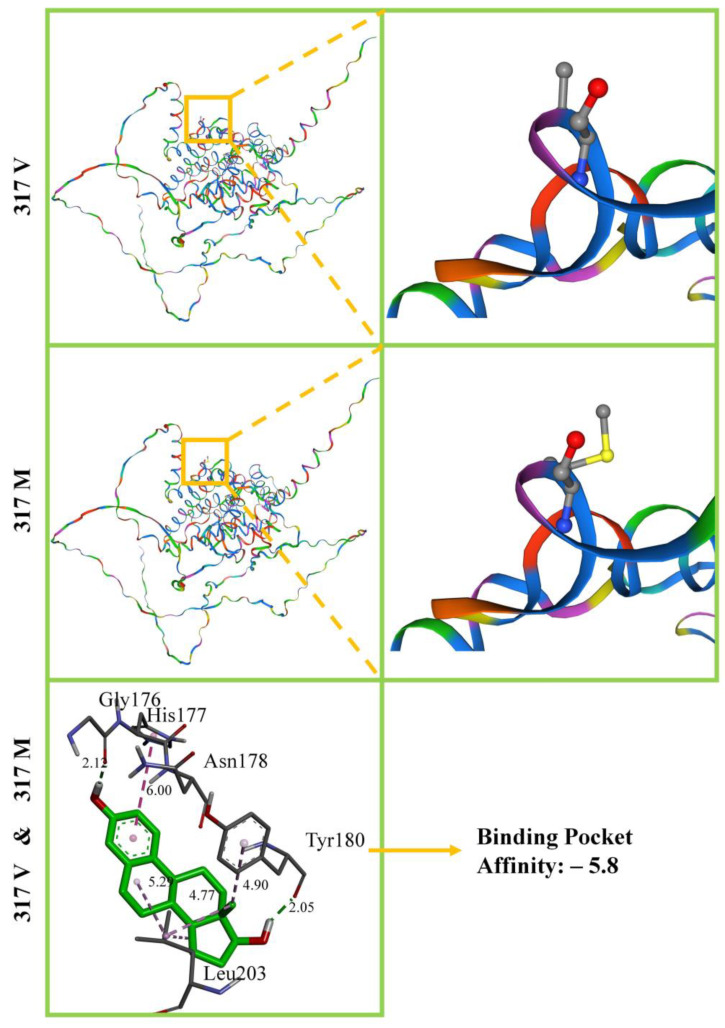
Homology modelling of ESR’s 3D structures and E_2_ docking. Moreover, 317V and 317M represent V>M mutations, with the orange box showing local amplification. It can be seen that V and M are arranged at the 317th position on the amino acid chain. No spatial structural differences are evident, whether overall or local. Bottom of the figure is a partial magnification of the ESR and E_2_ docking binding pocket, excluding the 317th position on the amino acid chain. Bond unit: angstrom.

The *ESR* SNP 388G>A resulted in a 317V>M mutation in the amino acid chain. Figure 4 shows the ESR 3D structures of 317V and 317M, and there is no difference between them, while the spatial structure remains unchanged. The local structure magnified the positions at 317V and 317M, but no changes in the spatial structure were found. The results of docking with E_2_ using the 17V and 317M homology models were the same. We found binding pockets in Gly 176, His 177, Asn 178, Tyr 180, and Leu 203. The affinity of both was −5.8.

## 4. Discussion

The significance of studying swine LS is noteworthy as it not only increases the income of pig farms but also reduces the number of sows, contributing to reduced environmental pollution [58], and provides experimental animal models for human medical research [59]. The various meta-analysis results of this study indicate that *ESR* can indeed regulate LS in pigs, but there is no significant difference between low and high LSs related to *ESR* genes in the population. We believe that this result is due to unclear SNP positioning.

### 4.1. PCR-RFLP/SSCP Defects in Identifying SNPs

Multiple SNP loci exist simultaneously in a single gene [27]. In studies targeting different lines of LW, line B was treated with *Alu I* and *Hpa II*, achieving opposite results [18]. The same population can be divided into groups based on the SNPs of BF, *RBP4*, and *ESR2* [21]. A simultaneous evaluation of multiple genes in swine LS can yield more accurate results [22]. In reports on the regulation of LW LS by *ESR* and leptin, the impact of leptin is significant, and when grouped according to *ESR*, leptin masks some data [41]. Therefore, using PCR-RFLP/SSCP to separate individual gene SNPs into groups is flawed, leading to a decrease in the credibility of the results. In addition, the results of *ESR* 3D structures and E_2_ docking in this study also showed that the impact of *ESR* SNP 388G>A on LS was limited, and the reliability of the results based on such SNPs for sample sow grouping was also limited.

### 4.2. GWAS Defects in Identifying SNPs

The following have been observed when identifying SNPs: (i) The frequency of key genes in the population reached 100%. *ESR* (1227 C>T) and *FSH* (930 A>G) are already homozygous in swine production farms [38], and SNPs are reported when analysed using the software. (ii) GWASs failed to select a suitable chip. Some studies have focused on different pig breeds but used the same SNP chip, resulting in inconsistent results [23]. (iii) Individual records are not comprehensive. Disease [10], stress, gut microbiota [14], and adding antibiotics to feed can affect the immune response. Diseases can alter immune genes in swine populations [60].

The above factors can affect LS, so SNPs in immune-related genes were selected for GWAS research. When collecting experimental individuals, we usually choose a group based on their consistent genetic background. Pigs recovered from minor illnesses were not recorded on the pig farm, based on the above results. Salmonella infection remains a major problem in pig farms [61], and in Germany, over 90% of pig farms tested positive [62]. Therefore, this study used GWAS candidate gene enrichment analysis to identify immune stress genes as the key genes. The genes deposited in the database (www.animalgenome.org) need to be reviewed [3]; therefore, immune-related genes have not been confirmed to be related to LS. Gene enrichment analysis was used to identify different pathways. The GWAS data collected in this study were all sourced from LW data, but only one *TRPC5* gene SNP was duplicated in the seven included studies.

### 4.3. Candidate Genes Affect Swine LS

There were more than seven studies on *ESR*, *PRLR*, *RBP4*, and *FSH β* related to swine LS. Unfortunately, most of these studies used the PCR-RFLP/SSCP method. There have also been reports on accurately locating SNPs. It has been reported that *FSH β* short interspersed nuclear elements (SINEs) and 212T>C both affect pig LS [24]. The *PRLR* SINE also affects pig LS [29]. Another study reported that *GDF9* affects swine LS and identified 12 SNPs, of which 3 are associated with LS [27]. Notably, *GDF9* 395 S>F and 427 S>R in sheep lead to an increase in ovulation rate due to heterozygous mutations, whereas homozygous mutations lead to infertility [63,64], which is of great significance for molecular-level animal breeding. This study used traditional meta-analysis to show that all four genes are positively correlated with pig LS. The network meta-analysis results showed no significant difference in the strength of the impact of these genes on LS. The genotype distribution results in the population indicate that *ESR*, *PRLR*, and *RBP4* high-LS genotypes do not dominate the population. Notably, if *FSH β* accurate localisation of SNPs is performed by removing re-statistics [24], *FSH β* genotypes also do not occupy a significant advantage in the population. The TSA results showed that, after 30 years of research, genotype classification using the PCR-RFLP/SSCP method did not reach a confidence interval. We believe that this result was due to unclear SNP localisation using the PCR-RFLP/SSCP method.

### 4.4. Relationship between LS and Litter Weight

Doom’s pig research showed that with a high LS, the total weight of piglets (litter weight) is low [25]. The study shows that Indian indigenous pigs with a high LS have a higher litter weight than low-LS pigs. [24]. A high litter weight indicates high uterine receptivity, but the article does not mention whether the body weight of high-LS sows is higher than that of low-LS sows. We propose a measurement method that considers the number of offspring produced under the same body weight conditions. Reproduction bearing capacity refers to the foetal total weight that a single or multiple individual animals can provide.

### 4.5. Lactation 

The results of this traditional meta-analysis indicated that *ESR*, *PRLR*, *RBP4*, and *FSH-β* determine LS and litter weight. However, the effects of these four genes were limited to a few days, and when piglets reached the weaning period, these effects were no longer significant. Thus, lactation is the key, and a high LS is associated with lower lactation than a low LS. Doom’s pigs in the high-LS group had a lower birth weight than those in the low-LS group, and there was no significant difference in weaning weight, indicating that low-LS sows have higher lactation yields [25]. Based on our research, only two studies have reported pig lactation [1,28]. We estimated this by evaluating the difference between weaning and birth litter weights. We have been engaged in pig breeding and management for 20 years, and the pig farm that we investigated did not use artificial lactation. Currently, the only research on adding ingredients during piglet lactation involves artificial lactation [65]. Artificial breeding is a co-evolutionary process in which animals who meet human needs reproduce. Under the stressful conditions of artificial breeding, farms need to strive to achieve high commercial value. On the one hand, sows with high LS are kept in production. On the other hand, a lower milk yield can shorten the breeding cycle. Only sows that meet both of these requirements can survive.

### 4.6. Limitations

This study defines LS data as low and high, and the extracted datasets are the absolute values of LS changes, which may result in false-positive results. Because the purpose of the study is only to demonstrate that *ESR*, *PRLR*, *RBP4*, and *FSH β* are related to LS, this assumption is acceptable.

The *ESR* 3D structures have not been validated by X-ray crystal diffraction, and it is uncertain whether changes in individual amino acids affect *ESR* entry into the nucleus. The SNPs present in the introns were not analysed in this study. SNPs in introns may play a role in the transcription process and affect the regulation of variable splicing. This study has certain limitations. Only the GWAS methodology was reviewed in this study. However, the gold-standard genomic prediction method (GBLUP) was not covered. This is because while there is an abundance of literature related to GBLUP, GWAS is the most commonly used method in SNP research.

We did not delve into other potential reasons why high-LS genes in the population could not have a significant advantage. First, reproduction-related genes may be the opposite of those involved in meat production. The *ESR* gene regulates LS in Czech LW while also affecting lean meat percentage and average daily gain [35]. A high LS indicates a large weight of the uterus, many nipples, and a large weight of breast tissue. If a sow’s body weight is a fixed value, this may decrease meat production. Second, LS is a complex trait regulated by multiple genes. Our analysis shows that multiple genes can regulate LS, and selective breeding for a single gene is not appropriate.

### 4.7. Effects of the Development Trend of LS on Pig Breeding

Although we have discovered the shortcomings of PCR-RFLP/SSCP in accurately locating SNPs, this cannot negate the contributions of previous PCR-RFLP/SSCP research. The results of this study were based on numerous PCR-RFLP/SSCP studies, so we would like to thank the many PCR-RFLP/SSCP reporting research teams for their efforts. Further in-depth research should be conducted on the precise positioning of SNPs in the future [27]. An epigenome–transcriptome analysis is the current development direction [66], and LS research on the effects of porcine SNPs should also be combined with a conjoint analysis of the genome, epigenome, and transcriptome. The impact of immune response genes can be eliminated during GWAS analysis.

**Figure 5:**
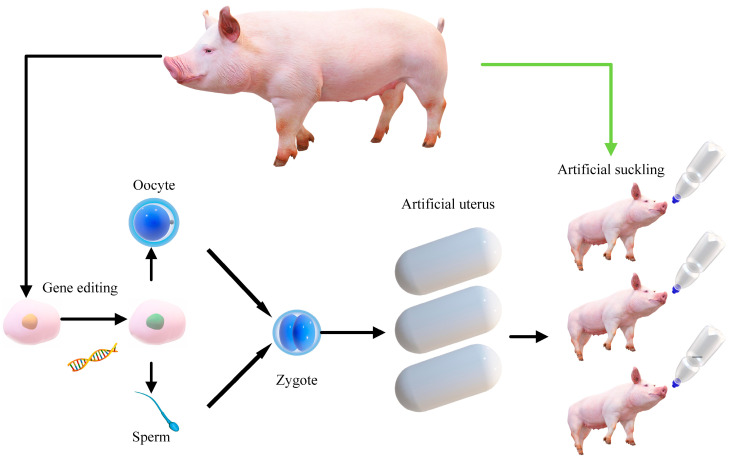
Futuristic piglet breeding system. Gene-edited somatic cells, which serve as the initial vectors, were induced into oocytes and sperm, respectively. After fertilization, they produced zygotes, which were cultured in an artificial uterus until birth. The piglets then underwent artificial lactation. The entire process did not involve breeding sows.

Based on our research results, we propose suggestions for developing LS in pig breeding (Figure 5) and the genes that play a role in mouse embryonic development [67]. In the future, genes that play a role in pig pregnancy could be identified as molecular genetic markers for pig breeding. Foetal genes can be altered by gene editing. ESC can develop directly into embryos [68]. In other path choices, somatic cells can become oocytes [69] and sperm [70]. Zygotes can then be cultured in an artificial uterus [71]. After birth, piglets are artificially fed, and the pig farm no longer raises sows for breeding. Realistic guidance for current application research shows that laying hens have lost broodiness through years of artificial breeding. Therefore, the current breeding direction is to develop artificial milk-feeding machines for piglets. In addition, it is necessary to screen individuals with high LS and lactation to shorten the breeding cycle. Of course, all processes must consider animal welfare and reduce environmental pollution.

## 5. Conclusions and Future Directions

The limitations of PCR-RFLP/SSCP in accurately locating swine SNPs have led to low credibility in defining LS results using PCR-RFLP/SSCP. The repeatability of GWAS research reports is not good, the genetic background of experimental materials should also be fully considered in the future, and suitable chips should be designed. Establishing and improving audited public databases is an important idea. We hypothesize that the future direction of pig breeding involves the widespread use of artificial feeding machines for piglet lactation and the screening of individuals with high LS and low lactation to shorten the breeding cycle. The breeding aim for sows will focus solely on bearing piglets, rather than lactation, which can shorten the breeding cycle.

## Figures and Tables

**Table 1:** Characteristics of selected genome-wide association studies on Large White.

Reference	Chip	Number of LW	Available SNPs	Detected Genes	Threshold −log10 (*p*-Value)	Study and Year
[52]	Illumina Porcine SNP60 Bead Chip	2410 sows 3122 boars	39,450 SNPs	*PCIF1*, *LRRC27*, *CFAP46*, *KNDC1*, *ADAM8*, *CALY*, *ECHS1*, *MTG1*, *CYP2E1*	0.05	Brinke, 2020
[53]	Bead Chips	7017 sows 1669 boars	55,375 SNPs	*TRRAP*, *MID1*, *ACOT9*, *TRPC5*, *MAMLD1*	0.05	Chen, 2021
[54]	GeneSeek Custom 50K SNP Chip, GeneSeek Custom 80K SNP Chip, Porcine SNP60 Bead Chip	11,451 sows 781 boars	11,230 SNPs	*GANC*, *VPS39*, *NCR2*, *NRN1*, *CUL9*, *LMBR1*, *KLF14*, *UBE2H*, *AHCYL2*, *SMO*, *NCR2*, *FOXP4*, *MDFI*, *TRERF1*, *ADGRF1*, *SH3GL3*, *FSD2*, *AP3B2*, *PSTPIP1*, *ARID3B*, *FLT1*, *KATNAL1*, *USPL1*, *FRY*	≤6	Sell-Kubiak, 2022
[55]	Illumina Porcine SNP 60k Bead Chip	175 LW	47,865 SNPs	*ALDH1A3*, *LRRK1*, *TRPC5*, *RTL4*, *LHFPL1*, *SLITRK2*, *ALDH1A3*, *LRRK1*, *EPHB6*, *TRPC5*, *RTL4*, *LHFPL1*	NM	Suwannasing, 2018
[2]	50K chip	2655 LW	43,549 SNPs	*TTLL11*, *UNC93B1*, *TBX10*, *CDC14A*, *COL2A1*, *SENP1*, *CCDC184*, *KANSL2*, *SLC41A2*, *GSC*, *KLF3*, *PLXDC2*, *RIPK4*, *MMADHC*, *LYPD6*, *KIF5C*, *EPC2*, *ORC4*	0.05	Wang, 2022
[56]	Porcine SNP80 Bead Chip	1207 LW	51,443 SNPs	*IFITM2*, *IL1B2*, *GCK*	≤6	Wang, 2018
[57]	Affymetrix Porcine 55 K SNP Chip	695 LW	64,812 SNPs	*STT3B*, *THRB*, *TUSC3*	≤5	Wu, 2022

Abbreviations: not mentioned (NM).

## Data Availability

Please contact Zhenhua Guo for data requests.

## Supplementary Materials

The following supporting information can be downloaded at: https://www.mdpi.com/article/10.3390/cimb46070378/s1. Supplementary Table S1. Characteristics of studies selected of SNP. Refs. [1,2,16,17,18,19,20,21,22,23,24,25,26,27,28,29,30,31,32,33,34,35,36,37,38,39,40,41,42,43,44,45,46,47,48,49,50,51,72,73,74,75,76,77,78,79,80,81,82,83,84,85,86,87,88,89,90,91,92,93,94,95,96,97,98,99,100,101,102,103,104,105,106,107,108,109,110,111,112,113,114,115,116,117,118,119,120,121,122,123,124,125,126,127,128,129,130,131,132,133,134,135,136,137,138,139,140,141,142,143,144,145,146,147,148,149] are cited in Supplementary Materials file.

## Abbreviations

Circular RNA (circRNA), endocrine-disrupting chemicals (EDCs), genome-wide association studies (GWASs), long noncoding RNAs (lncRNAs), litter size (LS), Large White (LW), micro RNA (miRNA), polymerase chain reaction–restricted fragment length polymorphism (PCR-RFLP), polymerase chain reaction–single-strand conformational polymorphism (PCR-SSCP), protein–protein interaction (PPI), single-nucleotide polymorphisms (SNPs), short interspersed nuclear elements (SINEs), trial sequential analysis (TSA).
