# Supplementary material for: Effect of SNPs on Litter Size in Swine
Source: Curr Issues Mol Biol. 2024 Jun 24;46(7):6328–45. doi: 10.3390/cimb46070378 (PMC11276056; doi:10.3390/cimb46070378)
Supplement: Supplementary file 1 [file cimb-46-00378-s001.zip › cimb-2945041-Supplmentary.pdf]

Supplementary table 1. Characteristics of studies selected of SNP

Study and year	Breed	party	Gene	PCR-RFLP/SSCP	SNP endonuclease	TDN	NDN
**Alberto, 2009 [1]**	YOR, LAN, DUR	2	*PRLR*	Yes	*Hpa II*, *Alu I*	1	No
**An, 2017[2]**	BER	NM	*IGFBP2*, *IGFBP3*	Yes	*Mbo II*, *BsaH I*	No	No
**Balcells,2011 [3]**	IBE × MEI	NM	*MUC4*	No	No	No	No
**Balcells, 2011[4]**	IBE × MEI, LAN, LW, PIE	NM	*ITIH SSC13*	Yes	*Exonucease I*	No	No
**Buske, 2005[5]**	LW, LAN, LEI	more than 4	*BF*	Yes	*MAS*	No	No
**Buske, 2006[6]**	(LW × LAN) sows × LEI boars	NM	*ESR2*, *CYP21*	Yes	*Hsp92 II*, *Hae III*, *Pst I*	No	No
**Chen, 2001[7]**	YOR, LAN, ERH, XIAN	more than 1	*ESR*, *FSHβ*	Yes	*PVU II*	4	4
**Chen, 2004[8]**	LAN, YOR, DUR.	more than 4	*LEPR*	Yes	*EcoR I*	No	No
**Coster, 2012)[9]**	LW	4	*DIO3*	No	No	No	No
**Dall'Olio, 2010[10]**	Italian LW	NM	*ESR2*, *AFP*, *BMPRIB*, *CLGN*, *CXCL10*, *GNRHR*, *MAN2B2*, *RNF4*, *pDAZL*, *RBP4*	No	No	No	No
**Dan and Yonggang, 2020[11]**	LW, LAN, SAB, TIB, MING, DIA, WUJ, BAO	more than 7	*KLK7*	Yes	*Alu I*	No	No
**Depuydt, 1999[12]**	NM	2	*ESR*	No	No	No	No
**Ding, 2006[13]**	ERH x DUR	NM	*PTGS2*	Yes	*Mse I*	No	No
**Drogemuller, 2001[14]**	German LAN, DUR, LW	10	*ESR*, *PRLR*, *RBP4*	Yes	*Pvu II*, *Alu I*, *Msp I*	No	No
**Du, 2009[15]**	DUR, ERH, DHB, LAN	NM	*ACVR1*, *ARHE*, *CED-6*, *CMYA3*, *COL4A4*, *DPP4*, *EPHA4*, *FLJ11457*, *GAD1*, *HAT1*, *HES6*, *HSPE1*, *KCNJ3*, *KLF7*, *NFE2L2*, *NR4A2*, *NRP2*, *ORC2L*, *SH3BP4*, *SP3*, *TRIP12*	Yes	*Cfr I*, *Xap I*, *BseM I*, *Eco105 I*, *Bsh1236 I*, *Tas I*	No	No
**Du, 2022[16]**	TIB, WUZ, BAMA, DUR, HAM, BER, PIE, LAN, YOR, BAME, JIN, RON, MEI,	1	*IDO2*, *OPN PRLR*, *CWH43*, *VMP1*,	No	No	1	No
**Fang, 2014[17]**	YOR, LAN, DUR, LANT, SES	6	*RFRP*, *β-actin*	Yes	NM	No	No

**Feng, 2013[18]**	LAN, LW, DUR	NM	*BMP-7*	Yes	*Ava I*	No	No
**Fernández-Rodríguez, 2010 [19]**	IBE MEI	NM	*SLC9A3R1*, *NOS2*	Yes	*MAS, GAS*	No	No
**Franco, 2005[20]**	LAN	NM	*PIT1*, *GH*, *GHRH*	Yes	*RsaI*, *Dde I*, *Alu I*	No	No
**Gibson, 2002[21]**	MEI, LW	2	*ESR*	Yes	*Pvu II*	No	No
**Goliasova and Wolf, 2004[22]**	Czech LW	1	*ESR*	Yes	*Pvu II*	1	No
**Gonçalves, 2008[23]**	Commercial sows	4	*ER*, *RBP4*	Yes	*Pvu II*, *Msp I*	2	No
**He, 2017[24]**	ERH	7	*UCHL1*, *RPS6KB1*, *CLTC*	No	No	No	No
**He, 2021[25]**	DUR sows	NM	*EXOC4*	Yes	*Hind III*, *MIu I*	No	No
**Horak, 2005[26]**	Prestice Black-Pied sows	NM	*FUT1*, *ESR2*	Yes	*Pvu II*	1	No
**Horogh, 2005[27]**	Hungarian LW	more than 3	*ESR*	Yes	*Pvu II*	3	No
**Houde, 2008[28]**	LAN, DUR, YOR, MEI–LAN.	10	*ADIPOQ*, *ADIPOR1*, *DIPOR2*	Yes	*Superscipt II*	No	No
**Hunyadi-Bagi, 2016[29]**	Hungarian LW, DUR, PIE	1	*BF*, *EGF*, *ESR*, *FSH B*, *H2AFZ*, *LEP*, *PRLP*	Yes	NM	No	No
**Hwang, 2018[30]**	BER	more than 1	*NAT9*, *MAP3K3*	Yes	*Alu I*, Xcm I	No	No
**Jafarikia, 2015[31]**	DUR, LAN, YOR	1-10	*ADIPOQ*	Yes	NM	No	No
**Jiugang, 2012[32]**	LW, LAN, SAB, TIB, MING, DIA, WUJ, BAO	more than 7	*NAT9*, *GAPDH*	Yes	*Alu I*	No	No
**Kumchoo and Mekchay 2015[33]**	LW, LAN	2	*NR4A1; GNB2L1;*	Yes	*Dde I*, *Taq I.*	No	No
**Laliotis, 2017[34]**	Greek black autochthonous pig	5	*BF*, *RBP4*, *ESR2*	Yes	*Msp I*, *Hsp92 II*, *Sma I*	2	2
**Lan, 2012[35]**	LW, LAN, SAB, TIB, MING, DIA, WUJ, BAO	more than 7	*ROPN1*	Yes	*Hae III*	No	No
**Laplana, 2020[36]**	LAN LW sows	more than 3	*SGK1*, *TAP1*	No	No	No	No
**Lei and Yonggang 2012[37]**	LW, LAN, SAB, TIB, MING, DIA, WUJ, BAO	more than 7	*OSAP*	Yes	*Xba I*	No	No
**Li, 2002[38]**	ERH, Lingao, MEI, DUR, LW, LAN, DW2 line, LWXM, TON, OIN, BAME	3	*FSHβ*	NM	NM	3	No

**Lin, 2006[39]**	PIE x HAM	NM	*β-actin (ACTB)*	No	No	No	No
**Lin, 2009[40]**	LW	8	*LIT*	No	No	No	No
**Linville, 2001[41]**	LAN, LW	NM	*ESR*, *EGF*, *RBP4*, *PTGS2*, *FSHβ*, *PRLR*	Yes	*Pvu II*, *Msp I*, *Mse I*, *ATaq I*, *Alu I*	No	No
**Liu, 2018[42]**	XIAN	NM	*PDIA4*	NM	NM	No	No
**Liu, 2015[43]**	LW, LAN, SAB, TIB, MING, DIA, WUJ, BAO	more than 7	*GADD45G*	Yes	*Rsa I*	No	No
**Liu, 2016[44]**	TON, MEI, LWi, LW II	more than 4	*ALAS1*	Yes	*Msp I*	No	No
**Liu, 2011[45]**	TON, MEI, LW, DIV	more than 4	*NR4A1*	Yes	*Dde I*	No	No
**Liu, 2009[46]**	LAN, TON, HUAI, MEI, LW	more than 4	*HSD17B1*	Yes	*Mva I* *Nde I*	No	No
**Liu, 2019[47]**	YOR, Yimeng black pigs	more than 1	*NRDR*	No	No	No	No
**Magotra 2015[48]**	Niang Megha, Ghungroo pigs	NM	*FSH β*	Yes	SINE	2	No
**Marantidis, 2016[49]**	LW × LAN	3	*RBP4*	Yes	*Msp I*	3	No
**Marantidis, 2013[50]**	LW x LAN	more than 5	*BF*	Yes	*Sma I*,	No	No
**Marek, 2001[51]**	Polish LAN	1	*PRLR*	Yes	*Alu I*	1	No
**Menčik, 2020[52]**	LAN x LW	6	*RNF4*	Yes	*Sac II*	No	No
**Menčik, 2019[53]**	LAN × LW	1	*ESR1*, *RBP4*	Yes	*Pvu II.*, *Msp I.*	6	6
**Mihailov, 2014[54]**	LW, LAN, LAN × YOR × DUR.	NM	*PRLR*	Yes	*Alu I*	2	No
**Mucha, 2013[55]**	Polish LW, LAN	8	*EGF*, *AREG*, *LIT*	Yes	*Sty I Dra III*	No	No
**Muñoz, 2007)[56]**	MEI, Jiaxing pig, LW, SpanishWild boars	NM	*ESR1*, *ESR2*	Yes	*Ava I*, *Pvu II*, *Hsp92 II*	No	No
**Muñoz, 2010[57]**	Chinese-European porcine line	more than 6	*RBP4*, *ESR1*, *IGF2*	Yes	*Msp I*, *Pvu II*	No	No
**Naha, 2020[58]**	LAN, Indigenous pigs	NM	*ESR1*, *Prei3*, *FSHβ*, *OPN*, *CDK20*	Yes	NM	No	No
**Niu, 2013[59]**	LAN, LW, TON, MEI	4	*pMMP-9*	Yes	*Msp I*, *Sma I*,	No	No
**Niu, 2008[60]**	TIB	more than 8	*RBP4*, *OPN*	Yes	*Msp I*	2	No
**Noguera, 2003[61]**	LAN	more than 3	*ESR*	Yes	*Pvu II*	2	No

**Norseeda, 2021[62]**	LAN	1至8	*IL-4*, *IL-4R*	Yes	*BsuR I*, *PflF I*, *fl III*, *Mlu I*, *BsuR I*, *Alu I*	No	No
**Omelka, 2008[63]**	LW, White Meaty, LAN	1	*PRLR*	Yes	*Alu I*	3	No
**Panasiewicz, 2017[64]**	Hirschmann hybrid-line sows	8	*family pPAGs*	Yes	*Mnl I*, *Alu I*, *BstKT I*	No	No
**Pang, 2019[65]**	LW sows	more than 4	*FSHβ*, *ESR*, *CTNNAL1*, *miR-27a*	Yes	*Pvu II*, *Alu I*, *Hpa II*	2	2
**Pradhan, 2018[66]**	Ghoongroo sow	NM	*B*	NM	NM	No	No
**PutnovÁ 2002[67]**	LAN, LW	NM	*PRLR*	Yes	*Alu I*	6	No
**Rahman, 2021 [68]**	Doom Pigs	NM	*ESR*	Yes	*Alu I*	1	No
**Rempel, 2010[69]**	LAN-DUR-YOR.	NM	*PRLR*, *ESR*	No	No	No	No
**Rothschild, 1996[70]**	MEI, LW	more than 1	*ER*	Yes	*Pvu II*	No	No
**Rothschild, 2000[71]**	LW, LAN, DUR	NM	*RBP4*	Yes	*Msp I*	2	No
**Santana, 2006[72]**	LW, LAN, PIE	NM	*ESR*	Yes	*Pvu II*	No	No
**Shife and Yonggang i, 2012[73]**	LW, LAN, SAB, TIB, MING, DIA, WUJ, BAO	more than 7	*IBP4*	Yes	*Hha I*	No	No
**Sironen, 2010[74]**	Finnish YOR, LAN	1	*CPT1A*, *IGFBP1*, *IGFBP3*, *SLC22A5*, *COX2*, *IGFBP2*, *IGFBP5*	Yes	*BstN I*, *BtsC I*, *BsaH I*, *Hae III*, *BsrB I*, *Mbo II*, *Ava I*	No	No
**Spötter, 2005[75]**	synthetic German pig	10	*LIF*	Yes	*Dra III*	No	No
**Spotter, 2009[76]**	LW, German LAN	2-3	*LIF*, *RBP4*	Yes	*Dra III* *Msp I*	4	4
**Sven2015[77]**	NM	3	*PRLR*	Yes	*Alu I*	3	No
**Tao, 2013[78]**	Taihu pigs, TON, HEZ, HUAI, DIV, LW, LAN, DUR, PIE	more than 4	*TCF12*, *CTNNAL1*, *WNT10B*	Yes	*Mbi I*, *BsrG I*, *Alu I*, *Sac II*	No	No
**Tempfli, 2011[79]**	Mangalica	NM	*PRLR*	Yes	*Alu I*	1	No
**Terman, 2005[80]**	LW × LAN	4	*PRLR*, *LEP*	Yes	*Alu I*, *Hinf I*	4	4
**Terman, 2011[81]**	LW × LAN	1	*RBP4*	Yes	*Msp I*	3	No
**Terman and Kumalska, 2012[82]**	Polish LW, LAN	more than 3	*ESR*	Yes	*Ava I*	6	No
**Terman, 2016[83]**	Polish swine	3	*PRLR*	Yes	*Alu I*	3	No

**Tomás, 2006)[84]**	IBE × MEI	4	*PRLR*	NM	NM	No	No
**Vallet, 2005[85]**	MEI, YOR, LAN, DUR	NM	*EPOR*	No	No	No	No
**Vallet, 2005[86]**	MEI-LW	NM	*sFBP*	No	No	No	No
**van Rens, 2003[87]**	LAN, MEI	NM	*PRLP*	Yes	*Alu I*	No	No
**Vashi, 2021[88]**	NM	1	*ESR*, *EGF*, *FSHβ*, *PRLR*, *RBP4*	Yes	*Pvu II*, *Alu I*, *Msp I*	4	4
**Vega, 2018[89]**	LW sows	NM	*LEP*, *ESR*	Yes	*Pvu II*	1	No
**Wang, 2012[90]**	LW, LAN, Saba, Tibetan, MING, DIA, WUJ, BAO)	more than 7	*SPATA19*	Yes	*Alu I-*	No	No
**Wang, 2008[91]**	BB	NM	*PRLR*, *BF*	Yes	*Alu I*, *Sma I*	2	2
**Wu, 2019[92]**	Jiaxing Black sows	2	*GPR54*	No	No	No	No
**Wu, 2010[93]**	LW sows	2	*TGF-b1*	No	No	No	No
**Xiao, 2012[94]**	LW, LAN, DUR	9	*Muc1*	No	No	No	No
**Xudong, 2017[95]**	LW, LAN, SAB, TIB, MING, DIA, WUJ, BAO	more than 7	*HTRA3*	Yes	*Hpa II*	No	No
**Yin, 2019[96]**	LW	NM	*BMP7*, *BMP*	Yes	NM	No	No
**Yong, 2012[97]**	LW, LAN, SAB, TIB, MING, DIA, WUJ, BAO	more than 7	*MYST2*	Yes	*Pst I*	No	No
**Yonggang and Xueshan2012[98]**	LW, LAN, SAB, TIB, MING, DIA, WUJ, BAO	more than 7	*LCK*	Yes	*Alu I-*	No	No
**Yuan, 2007[99]**	MEI, OIN, JIN, DUR, LW, LAN	more than 2	*FSHR*, *ZP3*	No	No	No	No
**Yuan, 2007[100]**	MEI, OIN, DUR, LAN, LW	more than 2	*ZP3*	Yes	*Rsa I*, *BstF5 I*, *Mse I*, *Taq I*, *Fnu4H I*, *Bcc I*, *Hinf I*, *HpyCH4 III*	No	No
**Yurina, 2021[101]**	LW	NM	*ESR*<br>*PRLR*, *FSHβ*	Yes	*Pvu II*<br>*Alu I*<br>*BsuR I*	3	No
**Žákováand Wolf, 2004[102]**	Czech LW sows	more than 2	*ESR*	Yes	*Pvu II*	2	No
**Zhang, 2011[103]**	BB	more than 1	*EPOR*	No	No	No	No
**Zhang, 2019[104]**	NM	more than 1	*AHR*	No	No	No	No

**Zhang, 2012[105]**	XIAN, LANT, SES, Zang pig, WUJ, Gaoligongshan pig, BAO, Sakan pig, LAN, YOR, DUR	6	*TDRP1*, *β-actin*	Yes	*Ssp I*	No	No
**Zhang, 2015[106]**	Wannan Black pigs, BW, BER	2	*FSHR*	Yes	No	No	No
**Zhang, 2008[107]**	DUR, ERH, DHB, LAN	NM	*GDF9*	Yes	*Mse I*, *Dde I*, *Hinf I*	No	No
**Zhang, 2009[108]**	LW, LAN, MEI, BAME, HUAI, TON, HEZ, DIV	more than 4	*pDAZL*	Yes	*Msp I*, *Taq I*	No	No
**Zhang, 2009[109]**	MEI, HEZ, TON, HUAI, BAME, LW, DIV, LAN, LW	more than 4	*H2AFZ*	Yes	*Bsu15 I*	No	No
**Zhao, 1998[110]**	ERH, Minipig, LAN, YOR	4	*FSHβ*	Yes	NM	No	No
**Zhao and Liu, 2017[111]**	LW, LAN, Saba, TIB, MING, DIA, WUJ, BAO	more than 7	*SUN5*	Yes	*Taq I*	No	No
**Zhu, 2017[112]**	Hebao, DUR, LAN, LW	NM	*ESR*, *FSHβ*, *MC4R*, *A-FABP*	Yes	*Pvu II*, *Taq I*, *Bsm I*, *Hinf I*, *Hae III*	8	8
**Zhu, 2004[113]**	TON	1	*ESR*	Yes		No	No
**Ziółkowska, 2010[114]**	Polish LW, LAN	NM	*PRLR*	Yes	*Alu I*, *Msp I*	No	No

Abbreviations: Not mentioned (NM). Traditional meta-analysis dataset number (TDN). Network meta-analysis dataset number (NDN). Bama pig (BAMA). Bamei pig (BAME). Baoshan pig (BAO). Beijing Black pig (BB). Berkshire (BER). Dahuabai pig (DHB). Diannan small-ear pig (DIA). Duroc (DUR). Erhualian (ERH). Hampshire (HAM). Hezuo pig (HEZ). Huainan pig (HUAI). Iberian (IBE). Landrace (LAN). Lantang (LANT). and Leicoma (LEI). Large White (LW). Jinhua pig (JIN). Meishan pig (MEI). Mingguang small-ear pig (MING). Pietrain (PIE). Qingping pig (QIN). Rongchang (RON). Saba pig (SAB). Small-ear spotted pig (SES). Tibetan pig (TIB). Tongcheng (TON). Wujin pig (WUJ). Wuzhishan pig (WUZ). Xiang pig (XIAN). Yorkshire (YOR).

Reference

1. Alberto, B.S.; Haro, J.; Hori-Oshima, S.; Espinosa, A.; Ortega, M.; Perez, J.; Lemus-F., C.; Espinosa, A.L.; Aranguré, A.; Avila, J.G. Prolactin Receptor (PRLR) Gen Polymorphism and Associations with Reproductive Traits in Pigs. *Journal of Animal and Veterinary Advances* **2009**, *8*, 469-475.
2. An, S.; Hwang, J.; Kwon, S.; Yu, G.; Park, D.; Kang, D.; Kim, T.; Park, H.; Ha, J.; Kim, C. Effect of Single Nucleotide Polymorphisms in IGFBP2 and IGFBP3 Genes on Litter Size Traits in Berkshire Pigs. *Animal Biotechnology* **2017**, *29*, 1-8, doi:10.1080/10495398.2017.1395345.

3. Balcells, I.; Castelló, A.; Mercadé, A.; Noguera, J.L.; Fernández-Rodríguez, A.; Sànchez, A.; Tomàs, A. Analysis of porcine MUC4 gene as a candidate gene for prolificacy QTL on SSC13 in an Iberian × Meishan F2 population. *BMC Genet* **2011**, *12*, 93, doi:10.1186/1471-2156-12-93.
4. Balcells, I.; Castelló, A.; Noguera, J.L.; Fernández-Rodríguez, A.; Sánchez, A.; Tomás, A. Sequencing and gene expression of the porcine ITIH SSC13 cluster and its effect on litter size in an Iberian × Meishan F2 population. *Animal reproduction science* **2011**, *128*, 85-92, doi:10.1016/j.anireprosci.2011.09.004.
5. Buske, B.; Brunsch, C.; Zeller, K.; Reinecke, P.; Brockmann, G. Analysis of properdin (BF) genotypes associated with litter size in a commercial pig cross population. *Journal of Animal Breeding and Genetics* **2005**, *122*, 259-263, doi:https://doi.org/10.1111/j.1439-0388.2005.00528.x.
6. Buske, B.; Sternstein, I.; Reißmann, M.; Brockmann, G. Detection of novel single-nucleotide polymorphisms (SNPs) in the CYP21 gene and association analysis of two SNPs for CYP21 and ESR2 with litter size in a commercial sow population. *Journal of Animal Breeding and Genetics* **2006**, *123*, 343-348, doi:https://doi.org/10.1111/j.1439-0388.2006.00609.x.
7. Chen, K.; Li Barr, N.; Huang, L.; Zhang, Q.; Zhang, J.; Sun, S.; Luo, M.; Wu, C. The combined genotypes effect of ESR and FSH?? genes on litter size traits in five different pig breeds. *Chinese Science Bulletin* **2001**, *46*, 140-143, doi:10.1007/BF03187010.
8. Chen, C.C.; Chang, T.; Su, H.Y. Characterization of porcine leptin receptor polymorphisms and their association with reproduction and production traits. *Animal biotechnology* **2004**, *15*, 89-102, doi:10.1081/abio-120037903.
9. Coster, A.; Madsen, O.; Heuven, H.; Dibbits, B.; Groenen, M.; Arendonk, J.; Bovenhuis, H. The Imprinted Gene DIO3 Is a Candidate Gene for Litter Size in Pigs. *PloS one* **2012**, *7*, e31825, doi:10.1371/journal.pone.0031825.
10. Dall'Olio, S.; Fontanesi, L.; Tognazzi, L.; Russo, V. Genetic structure of candidate genes for litter size in Italian Large White pigs. *Veterinary Research Communications* **2010**, *34*, 203-206, doi:10.1007/s11259-010-9380-7.
11. Dan, L.; Yonggang, L. Molecular characteristics and association analysis with litter size trait for porcine KLK7 gene. *Animal biotechnology* **2020**, *31*, 377-381, doi:10.1080/10495398.2019.1604379.
12. Depuydt, J.; De Smet, S.L.A.X.; Grijspeerdt, K.; Herman, L. Association study of an AvaI and PvuII polymorphism at the porcine estrogen receptor (ESR) gene, with litter size. *(1999) ARCHIV FUR TIERZUCHT-ARCHIVES OF ANIMAL BREEDING* **1999**.

13. Ding, N.-S.; Ren, D.-R.; Guo, Y.-M.; Ren, J.; Yan, Y.; Ma, J.-W.; Chen, K.-F.; Huang, L.-S. Genetic variation of porcine prostaglandin-endoperoxide synthase 2 (PTGS2) gene and its association with reproductive traits in an Erhualian x Duroc F2 population. *Yi Chuan Xue Bao* **2006**, *33*, 213-219, doi:10.1016/s0379-4172(06)60042-5.

14. Drogemuller, C.; Hamann, H.; Distl, O. Candidate gene markers for litter size in different German pig lines1. *Journal of Animal Science* **2001**, *79*, 2565-2570, doi:10.2527/2001.79102565x.

15. Du, H.; Chen, J.; Cui, J.; Wang, X.; Zhang, X. Polymorphisms on SSC15q21-q26 Containing QTL for reproduction in Swine and its association with litter size. *Genetics and molecular biology* **2009**, *32*, 69-74, doi:10.1590/S1415-47572009000100010.

16. Du, Z.; D'Alessandro, E.; Asare, E.; Zheng, Y.; Wang, M.; Chen, C.; Wang, X.; Song, C. Retrotransposon Insertion Polymorphisms (RIPs) in Pig Reproductive Candidate Genes. *Genes (Basel)* **2022**, *13*, doi:10.3390/genes13081359.

17. Fang, M.X.; Huang, Y.S.; Ye, J.; Zhang, W.; Li, Y.; Nie, Q.H. Identification and characterization of RFRP gene in pigs and its association with reproductive traits. *Genet Mol Res* **2014**, *13*, 1661-1671, doi:10.4238/2014.january.14.8.

18. Feng, X.; Xie, S.-Y.; Zhou, J.-S.; Sun, G.-R.; Lu, P.; Li, M. Polymorphisms of the bone morphogenetic protein 7 gene (BMP7) and association analysis with sow productive traits. *Animal reproduction science* **2013**, *142*, 56-62, doi:10.1016/j.anireprosci.2013.08.011.

19. Fernández-Rodríguez, A.; Rodríguez, C.; Varona, L.; Balcells, I.; Noguera, J.L.; Óvilo, C.; Fernández, A.I. Analysis of candidate genes underlying two epistatic quantitative trait loci on SSC12 affecting litter size in pig. *Animal Genetics* **2010**, *41*, 73-80, doi:https://doi.org/10.1111/j.1365-2052.2009.01966.x.

20. Franco, M.; Antunes, R.; Silva, H.; Goulart, L. Association of PIT1, GH and GHRH polymorphisms with performance and carcass traits in Landrace pigs. *Journal of applied genetics* **2005**, *46*, 195-200.

21. Gibson, J.P.; Jiang, Z.H.; Robinson, J.A.B.; Archibald, A.L.; Haley, C.S. No detectable association of the ESR PvuII mutation with sow productivity in a Meishan × Large White F2 population. *Animal Genetics* **2002**, *33*, 448-450, doi:https://doi.org/10.1046/j.1365-2052.2002.00889.x.

22. Goliasova, E.; Wolf, J. Impact of the ESR gene on litter size and production traits in Czech Large White pigs. *Anim Genet* **2004**, *35*, 293-297, doi:10.1111/j.1365-2052.2004.01155.x.

23. Gonçalves, I.D.V.; Gonçalves, P.; Silva, J.C.; Jr, V.V.; Borges, L.F.K.; Oliveira, J.; Lovatto, P.A. Interaction between estrogen recepto and retinol-binding protein-4 polymorphisms as a tool for the selection of prolific pigs. *Genetics and Molecular Biology* **2008**, *31*, 481-486.

24. He, L.C.; Li, P.H.; Ma, X.; Sui, S.P.; Gao, S.; Kim, S.W.; Gu, Y.Q.; Huang, Y.; Ding, N.S.; Huang, R.H. Identification of new single nucleotide polymorphisms affecting total number born and candidate genes related to ovulation rate in Chinese Erhualian pigs. *Animal Genetics* **2017**, *48*, 48-54, doi:https://doi.org/10.1111/age.12492.

25. He, Y.; Zhou, X.; Zheng, R.; Jiang, Y.; Yao, Z.; Wang, X.; Zhang, Z.; Zhang, H.; Li, J.; Yuan, X. The Association of an SNP in the EXOC4 Gene and Reproductive Traits Suggests Its Use as a Breeding Marker in Pigs. *Animals : an open access journal from MDPI* **2021**, *11*, 521, doi:10.3390/ani11020521.

26. Horak, P.; Urban, T.; Dvorak, J. The FUT1 and ESR genes--their variability and associations with reproduction in Prestice Black-Pied sows. *J Anim Breed Genet* **2005**, *122*, 210-213, doi:10.1111/j.1439-0388.2005.00502.x.

27. Horogh, G.; Zsolnai, A.; Komiosi, I.; Nyiri, A.; Anton, I.; Fesus, L. Oestrogen receptor genotypes and litter size in Hungarian Large White pigs. *J Anim Breed Genet* **2005**, *122*, 56-61, doi:10.1111/j.1439-0388.2004.00483.x.

28. Houde, A.A.; Murphy, B.D.; Mathieu, O.; Bordignon, V.; Palin, M.F. Characterization of swine adiponectin and adiponectin receptor polymorphisms and their association with reproductive traits. *Animal genetics* **2008**, *39*, 249-257, doi:10.1111/j.1365-2052.2008.01714.x.

29. Hunyadi-Bagi, Á.; Balogh, P.; Nagy, K.; Kusza, S. Association and polymorphism study of seven candidate genes with reproductive traits in three pig breeds in Hungary. *Acta Biochim Pol* **2016**, *63*, 359-364, doi:10.18388/abp.2015_1188.

30. Hwang, J.H.; An, S.M.; Yu, G.E.; Park, D.H.; Kang, D.G.; Kim, T.W.; Park, H.C.; Ha, J.; Kim, C.W. Association of single-nucleotide polymorphisms in NAT9 and MAP3K3 genes with litter size traits in Berkshire pigs. *Arch Anim Breed* **2018**, *61*, 379-386, doi:10.5194/aab-61-379-2018.

31. Jafarikia, M.; Méthot, S.; Maignel, L.; Fortin, F.; Wyss, S.; Sullivan, B.; Palin, M.-F. Association of adiponectin and adiponectin receptor genes with sow productivity estimated breeding values. *Molecular biology reports* **2015**, *42*, 1391-1401, doi:10.1007/s11033-015-3917-3.

32. Jiugang, Z.; Jing, L.; Yonggang, L. Characterization, expression profile, polymorphism and association of porcine NAT9 gene. *Molecular biology reports* **2012**, *39*, 3137-3142, doi:10.1007/s11033-011-1079-5.

33. Kumchoo, T.; Mekchay, S. Association of NR4A1 and GNB2L1 genes with reproductive traits in commercial pig breeds. *Genetics and Molecular Research* **2015**, *14*, 16276-16284, doi:10.4238/2015.December.8.18.

34. Laliotis, G.P.; Marantidis, A.; Avdi, M. Association of BF, RBP4, and ESR2 Genotypes with Litter Size in an Autochthonous Pig Population. *Anim Biotechnol* **2017**, *28*, 138-143, doi:10.1080/10495398.2016.1242490.

35. Lan, J.; Zhao, J.; Liu, Y. Molecular cloning, sequence characterization, polymorphism and association analysis of porcine ROPN1 gene. *Molecular biology reports* **2012**, *39*, 2739-2743, doi:10.1007/s11033-011-1029-2.

36. Laplana, M.; Estany, J.; Fraile, L.J.; Pena, R.N. Resilience Effects of SGK1 and TAP1 DNA Markers during PRRSV Outbreaks in Reproductive Sows. *Animals : an open access journal from MDPI* **2020**, *10*, E902, doi:10.3390/ani10050902.

37. Lei, Y.; Yonggang, L. Cloning, sequence, and association analysis of porcine OSAP gene. *Appl Biochem Biotechnol* **2012**, *168*, 446-454, doi:10.1007/s12010-012-9788-3.

38. Li, F.; Yuanzhu, X.; Changyan, D.; Siwen, J.; Rong, Z. Frequencies, Inheritance of Porcine FSH-β Retroposon and its Association with Reproductive Traits. *Asian-Australasian Journal of Animal Sciences* **2002**, *15*, 179-183, doi:10.5713/ajas.2002.179.

39. Lin, C.L.; Jennen, D.G.J.; Ponsuksili, S.; Tholen, E.; Tesfaye, D.; Schellander, K.; Wimmers, K. Haplotype analysis of β-actin gene for its association with sperm quality and boar fertility. *Journal of Animal Breeding and Genetics* **2006**, *123*, 384-388, doi:https://doi.org/10.1111/j.1439-0388.2006.00622.x.

40. Lin, H.C.; Liu, G.F.; Wang, A.G.; Kong, L.J.; Wang, X.F.; Fu, J.L. Effect of polymorphism in the leukemia inhibitory factor gene on litter size in Large White pigs. *Molecular biology reports* **2009**, *36*, 1833-1838, doi:10.1007/s11033-008-9387-0.

41. Linville, R.C.; Pomp, D.; Johnson, R.K.; Rothschild, M.F. Candidate gene analysis for loci affecting litter size and ovulation rate in swine. *Journal of animal science* **2001**, *79*, 60-67, doi:10.2527/2001.79160x.

42. Liu, C.; Ran, X.; Niu, X.; Li, S.; Wang, J.; Zhang, Q. Insertion of 275-bp SINE into first intron of PDIA4 gene is associated with litter size in Xiang pigs. *Animal Reproduction Science* **2018**, *195*, 16-23, doi:https://doi.org/10.1016/j.anireprosci.2018.04.079.

43. Liu, C.; Zhang, W.; Yang, D.; Liu, Y. Molecular Characterization, Polymorphism, and Association of Porcine GADD45G Gene. *Animal biotechnology* **2015**, *26*, 230-236, doi:10.1080/10495398.2015.1005216.

44. Liu, L.Q.; Li, F.E.; Deng, C.Y. Short Communication: Molecular cloning and expression pattern of the porcine 5-aminolevulinate synthase 1 (ALAS1) gene and its association with reproductive traits. *Genet Mol Res* **2016**, *15*, doi:10.4238/gmr.15017544.

45. Liu, L.Q.; Li, F.E.; Deng, C.Y.; Xiong, Y.Z. Molecular cloning, tissue expression and association of porcine NR4A1 gene with reproductive traits. *Molecular biology reports* **2011**, *38*, 103-114, doi:10.1007/s11033-010-0083-5.
46. Liu, L.Q.; Li, F.E.; Deng, C.Y.; Zuo, B.; Zheng, R.; Xiong, Y.Z. Polymorphism of the pig 17beta-hydroxysteroid dehydrogenase type1 (HSD17B1) gene and its association with reproductive traits. *Animal reproduction science* **2009**, *114*, 318-323, doi:10.1016/j.anireprosci.2008.09.004.
47. Liu, Y.; Yang, Y.; Li, W.; Zhang, Y.; Yang, Y.; Li, H.; Geng, Z.; Ao, H.; Zhou, R.; Li, K. NRDR inhibits estradiol synthesis and is associated with changes in reproductive traits in pigs. *Molecular reproduction and development* **2019**, *86*, 63-74, doi:10.1002/mrd.23080.
48. Magotra, A.; Naskar, S.; Das, B.; Ahmad, T. A comparative study of SINE insertion together with a mutation in the first intron of follicle stimulating hormone beta gene in indigenous pigs of India. *Mol Biol Rep* **2015**, *42*, 465-470, doi:10.1007/s11033-014-3789-y.
49. Marantidis, A.; Laliotis, G.P.; Avdi, M. Association of RBP4 Genotype with Phenotypic Reproductive Traits of Sows. *Genet Res Int* **2016**, *2016*, 4940532, doi:10.1155/2016/4940532.
50. Marantidis, A.; Papadopoulos, A.I.; Michailidis, G.; Avdi, M. Association of BF gene polymorphism with litter size in a commercial pig cross population. *Animal Reproduction Science* **2013**, *141*, 75-79, doi:https://doi.org/10.1016/j.anireprosci.2013.06.011.
51. Marek, K.; Dybus, A.; Arkadiusz, T. Prolactin receptor gene polymorphism and its association with litter size in Polish Landrace. *Archiv fur Tierzucht* **2001**, *44*, 547-551, doi:10.5194/aab-44-547-2001.
52. Menčik, S.; Vuković, V.; Jiang, Z.; Ostović, M.; Sušić, V.; Žura Žaja, I.; Samardžija, M.; Ekert Kabalin, A. Effect of RNF4-SacII gene polymorphism on reproductive traits of Landrace × Large White crossbred sows. *Reproduction in domestic animals = Zuchthygiene* **2020**, *55*, 1286-1293, doi:10.1111/rda.13703.
53. Menčik, S.; Vukovic, V.; Spehar, M.; Modric, M.; Ostović, M.; Ekert Kabalin, A. Association between ESR1 and RBP4 genes and litter size traits in a hyperprolific line of Landrace × Large White cross sows. *Veterinární Medicína* **2019**, *64*, 109-117, doi:10.17221/87/2018-VETMED.
54. Mihailov, N.V.; Usatov, A.V.; Getmantseva, L.V.; Bakoev, S.U. Associations between PRLR/AluI gene polymorphism with reproductive, growth, and meat traits in pigs. *Cytology and Genetics* **2014**, *48*, 323-326, doi:10.3103/S0095452714050053.
55. Mucha, A.; Ropka-Molik, K.; Piórkowska, K.; Tyra, M.; Oczkowicz, M. Effect of EGF, AREG and LIF genes polymorphisms on reproductive traits in pigs. *Animal reproduction science* **2013**, *137*, 88-92, doi:10.1016/j.anireprosci.2012.12.009.

56. Muñoz, G.; Ovilo, C.; Estellé, J.; Silió, L.; Fernández, A.; Rodriguez, C. Association with litter size of new polymorphisms on ESR1 and ESR2 genes in a Chinese-European pig line. *Genet Sel Evol* **2007**, *39*, 195-206, doi:10.1186/1297-9686-39-2-195.

57. Muñoz, M.; Fernández, A.I.; Ovilo, C.; Muñoz, G.; Rodriguez, C.; Fernández, A.; Alves, E.; Silió, L. Non-additive effects of RBP4, ESR1 and IGF2 polymorphisms on litter size at different parities in a Chinese-European porcine line. *Genet Sel Evol* **2010**, *42*, 23, doi:10.1186/1297-9686-42-23.

58. Naha, B.; Gaur, G.; Saini, B.; Sahoo, N.; Boro, P. Polymorphism of candidate genes associated with reproductive performance in crossbred pigs. *The Indian Journal of Animal Sciences* **2020**, *90*, 809-812, doi:10.56093/ijans.v90i5.104637.

59. Niu, B.; Li, F.; Xiong, Y.; Wang, X. Characterization and association analysis with litter size traits of porcine matrix metalloproteinase-9 gene (pMMP-9). *Appl Biochem Biotechnol* **2013**, *171*, 786-794, doi:10.1007/s12010-013-0396-7.

60. Niu, S.; Wang, X.; Hao, F.; Zhao, R. Effect of the polymorphism of RBP4 and OPN genes on litter size in Tibet pigs. *Acta Agriculturae Scandinavica Section A-animal Science - ACTA AGR SCAND SECT A-ANIM SC* **2008**, *58*, 10-13, doi:10.1080/09064700802054170.

61. Noguera, J.L.; Varona, L.; Gomez-Raya, L.; Sanchez, A.; Babot, D.; Estany, J.; Messer, L.; Rothschild, M.; Pérez-Enciso, M. Estrogen receptor polymorphism in Landrace pigs and its association with litter size performance. *Livestock Production Science - LIVEST PROD SCI* **2003**, *82*, 53-59, doi:10.1016/S0301-6226(03)00004-6.

62. Norseeda, W.; Liu, G.; Teltathum, T.; Supakankul, P.; Sringarm, K.; Naraballobh, W.; Khamlor, T.; Chomdej, S.; Nganvongpanit, K.; Krutmuang, P.; et al. Association of IL-4 and IL-4R Polymorphisms with Litter Size Traits in Pigs. *Animals : an open access journal from MDPI* **2021**, *11*, 1154, doi:10.3390/ani11041154.

63. Omelka, R.; Martiniakova, M.; Peskovicova, D.; Bauerová, M. Associations between Alu I Polymorphism in the Prolactin Receptor Gene and Reproductive Traits of Slovak Large White, White Meaty and Landrace Pigs. *Asian-Australasian Journal of Animal Sciences* **2008**, *21*, doi:10.5713/ajas.2008.70314.

64. Panasiewicz, G.; Bieniek-Kobuszewska, M.; Lipka, A.; Majewska, M.; Jedryczko, R.; Szafranska, B. Novel effects of identified SNPs within the porcine Pregnancy-Associated Glycoprotein gene family (pPAGs) on the major reproductive traits in Hirschmann hybrid-line sows. *Res Vet Sci* **2017**, *114*, 123-130, doi:10.1016/j.rvsc.2017.03.015.

65. Pang, P.; Li, Z.; Hu, H.; Wang, L.; Sun, H.; Mei, S.; Li, F. Genetic effect and combined genotype effect of ESR, FSHbeta, CTNNAL1 and miR-27a loci on litter size in a Large White population. *Anim Biotechnol* **2019**, *30*, 287-292, doi:10.1080/10495398.2018.1486322.

66. Pradhan, M.; Pal, A.; Samanta, A.K.; Banerjee, S.; Samanta, R. Mutations in cytochrome B gene effects female reproduction of Ghungroo pig. *Theriogenology* **2018**, *119*, 121-130, doi:10.1016/j.theriogenology.2018.05.015.

67. Putnova, L.; Knoll, A.; DvoŘÁK, J.; ČEpica, S. A new HpaII PCR‐RFLP within the porcine prolactin receptor (PRLR) gene and study of its effect on litter size and number of teats. *Journal of Animal Breeding and Genetics* **2002**, *119*, 57-63, doi:10.1046/j.1439-0388.2002.00316.x.

68. Rahman, M.; Phookan, A.; Zaman, G.U.; Das, A.; Akhtar, F.; Tamuly, S.; Choudhury, H.; Sarma, L.M. Allelic variability of estrogen receptor (ESR) gene and its effect on litter traits of Doom pigs. *Trop Anim Health Prod* **2021**, *53*, 316, doi:10.1007/s11250-021-02756-6.

69. Rempel, L.A.; Nonneman, D.J.; Wise, T.H.; Erkens, T.; Peelman, L.; Rohrer, G.A. Association analyses of candidate single nucleotide polymorphisms on reproductive traits in swine. *Journal of animal science* **2010**, *88 1*, 1-15.

70. Rothschild, M.; Jacobson, C.; Vaske, D.; Tuggle, C.; Wang, L.; Short, T.; Eckardt, G.; Sasaki, S.; Vincent, A.; McLaren, D.; et al. The estrogen receptor locus is associated with a major gene influencing litter size in pigs. *Proc Natl Acad Sci U S A* **1996**, *93*, 201-205, doi:10.1073/pnas.93.1.201.

71. Rothschild, M.F.; Messer, L.; Day, A.; Wales, R.; Short, T.; Southwood, O.; Plastow, G. Investigation of the retinol-binding protein 4 (RBP4) gene as a candidate gene for increased litter size in pigs. *Mamm Genome* **2000**, *11*, 75-77, doi:10.1007/s003350010015.

72. Santana, B.; Biase, F.; Antunes, R.; Borges, M.; Franco, M.; Goulart, L. Association of the estrogen receptor gene Pvu II restriction polymorphism with expected progeny differences for reproductive and performance traits in swine herds in Brazil. *Genetics and Molecular Biology - GENET MOL BIOL* **2006**, *29*, doi:10.1590/S1415-47572006000200013.

73. Shifei, P.; Yonggang, L. Molecular characterization, polymorphism and association of porcine IBP4 gene. *Folia Biol (Praha)* **2012**, *58*, 30-36.

74. Sironen, A.I.; Uimari, P.; Serenius, T.; Mote, B.; Rothschild, M.; Vilkki, J. Effect of polymorphisms in candidate genes on reproduction traits in Finnish pig populations. *Journal of animal science* **2010**, *88*, 821-827, doi:10.2527/jas.2009-2426.

75. Spötter, A.; Drögemüller, C.; Hamann, H.; Distl, O. Evidence of a new leukemia inhibitory factor-associated genetic marker for litter size in a synthetic pig line1. *Journal of Animal Science* **2005**, *83*, 2264-2270, doi:10.2527/2005.83102264x.

76. Spotter, A.; Muller, S.; Hamann, H.; Distl, O. Effect of polymorphisms in the genes for LIF and RBP4 on litter size in two German pig lines. *Reprod Domest Anim* **2009**, *44*, 100-105, doi:10.1111/j.1439-0531.2007.01004.x.

77. Sven, M.; Vlado, V.; Mario, M.; Marija, S.; Mario, O.; Velimir, S.; Igor, S.; Marko, S.; Anamaria, E.K. Prlr-alui gene polymorphism and litter size traits in highly prolific line of topigs 20 sows. *Acta Veterinaria* **2015**, *65*, 463-476, doi:10.1515/acve-2015-0039.
78. Tao, H.; Mei, S.; Sun, X.; Peng, X.; Zhang, X.; Ma, C.; Wang, L.; Hua, L.; Li, F. Associations of TCF12, CTNNAL1 and WNT10B gene polymorphisms with litter size in pigs. *Animal reproduction science* **2013**, *140*, 189-194, doi:10.1016/j.anireprosci.2013.05.013.
79. Tempfli, K.; Farkas, G.; Simon, Z.; Bali Papp, A. Effects of prolactin receptor genotype on the litter size of Mangalica. *Acta Vet Hung* **2011**, *59*, 269-277, doi:10.1556/AVet.2011.012.
80. Terman, A. Effect of the polymorphism of prolactin receptor (PRLR) and leptin (LEP) genes on litter size in Polish pigs. *J Anim Breed Genet* **2005**, *122*, 400-404, doi:10.1111/j.1439-0388.2005.00547.x.
81. Terman, A.; Kmiec, M.; Polasik, D.; Rybarczyk, A. Association Between RBP4 Gene Polymorphism and Reproductive Traits in Polish Sows. *Journal of Animal and Veterinary Advances* **2011**, *10*, 2639-2641, doi:10.3923/javaa.2011.2639.2641.
82. Terman, A.; Kumalska, M. The effect of a SNP in ESR gene on the reproductive performance traits in Polish sows. *Russian Journal of Genetics* **2012**, *48*, 1260-1263, doi:10.1134/S1022795412120137.
83. Terman, A.; Polasik, D.; Korpal, A.; Wozniak, K.; Prüffer, K.; Żak, G.; Lambert, B. Association between prolactin receptor gene polymorphism (PRLR) with reproduction performance traits of Polish swine. *Canadian Journal of Animal Science* **2016**, *97*, doi:10.1139/CJAS-2016-0096.
84. Tomás, A.; Casellas, J.; Ramírez, O.; Muñoz, G.; Noguera, J.L.; Sánchez, A. High amino acid variation in the intracellular domain of the pig prolactin receptor (PRLR) and its relation to ovulation rate and piglet survival traits1. *Journal of Animal Science* **2006**, *84*, 1991-1998, doi:10.2527/jas.2005-664.
85. Vallet, J.L.; Freking, B.A.; Leymaster, K.A.; Christenson, R.K. Allelic variation in the erythropoietin receptor gene is associated with uterine capacity and litter size in swine. *Animal Genetics* **2005**, *36*, 97-103, doi:https://doi.org/10.1111/j.1365-2052.2005.01233.x.
86. Vallet, J.L.; Freking, B.A.; Leymaster, K.A.; Christenson, R.K. Allelic variation in the secreted folate binding protein gene is associated with uterine capacity in swine. *Journal of animal science* **2005**, *83*, 1860-1867, doi:10.2527/2005.8381860x.
87. van Rens, B.T.T.M.; Evans, G.J.; Lende, T.v.d. Components of litter size in gilts with different prolactin receptor genotypes. *Theriogenology* **2003**, *59*, 915-926, doi:https://doi.org/10.1016/S0093-691X(02)01155-X.

88. Vashi, Y.; Magotra, A.; Kalita, D.; Banik, S.; Sahoo, N.R.; Gupta, S.K.; Naskar, S. Evaluation of candidate genes related to litter traits in Indian pig breeds. *Reprod Domest Anim* **2021**, *56*, 577-585, doi:10.1111/rda.13895.

89. Vega, R.; Cho, B.-W.; Castillo, R.M.; Barrientos, N.; Autriz, M.; Viña, C. Leptin (T3469C) and Estrogen Receptor (T1665G) Gene Polymorphisms and Their Associations to Backfat Thickness and Reproductive Traits of Large White Pigs (Sus scrofa L.). *Philippine Journal of Science* **2018**, *14*.

90. Wang, J.Y.; Lan, J.; Zhao, J.; Chen, L.; Liu, Y. Molecular characterization, polymorphism and association of porcine SPATA19 gene. *Molecular biology reports* **2012**, *39*, 9741-9746, doi:10.1007/s11033-012-1839-x.

91. Wang, X.; Wang, L.; Zhuo-ma, L.U.O.; Shi-duo, S.U.N. Analysis of PRLR and BF Genotypes Associated with Litter Size in Beijing Black Pig Population. *Agricultural Sciences in China - AGRIC SCI CHINA* **2008**, *7*, 1374-1378, doi:10.1016/S1671-2927(08)60187-X.

92. Wu, F.; Zhang, W.; Song, Q.-Q.; Li, H.-H.; Xu, M.-S.; Liu, G.-L.; Zhang, J.-Z. Association analysis of polymorphisms of G protein-coupled receptor 54 gene exons with reproductive traits in Jiaxing Black sows. *Asian-Australas J Anim Sci* **2019**, *32*, 1104-1111, doi:10.5713/ajas.18.0827.

93. Wu, Y.P.; Wang, A.G.; Li, N.; Fu, J.L.; Zhao, X.B. Association with TGF-β1 Gene Polymorphisms and Reproductive Performance of Large White Pig. *Reproduction in Domestic Animals* **2010**, *45*, 1028-1032, doi:https://doi.org/10.1111/j.1439-0531.2009.01480.x.

94. Xiao, C.; Jinluan, F.; Aiguo, W. Effect of VNTR polymorphism of the Muc1 gene on litter size of pigs. *Molecular Biology Reports* **2012**, *39*, 6251-6258, doi:10.1007/s11033-012-1445-y.

95. Xudong, W.; Guiying, Z.; Guowen, F.; Yonggang, L. Molecular Cloning of the Porcine HTRA3 Gene and Association of a SNP with Litter Size Traits. *Folia Biol (Praha)* **2017**, *63*, 217-221.

96. Yin, H.; Du, X.; Li, Q.; Pan, Z.; Wu, W.; Liu, H.; Li, Q. Variants in BMP7 and BMP15 3'-UTRs Associated with Reproductive Traits in a Large White Pig Population. *Animals : an open access journal from MDPI* **2019**, *9*, E905, doi:10.3390/ani9110905.

97. Yong, W.J.; Jing, L.; Jiugang, Z.; Lei, C.; Yonggang, L. Molecular characterization, polymorphism and association of porcine MYST2 gene. *Molecular biology reports* **2012**, *39*, 7711-7716, doi:10.1007/s11033-012-1607-y.

98. Yonggang, L.; Xueshan, X. Molecular characterization, tissue expression, polymorphism and association of porcine LCK gene. *Molecular biology reports* **2012**, *39*, 4023-4028, doi:10.1007/s11033-011-1183-6.

99. Yuan, J.F.; Jafer, O.; Affara, N.A.; Gong, Y.Z.; Yang, L.G.; Liu, J.; Moaeen-ud-Din, M.; Li, W.M.; Zhang, S.J. Association of four new single-nucleotide polymorphisms in follicle-stimulating hormone receptor and zona pellucida glycoprotein with reproductive traits in pigs. *animal* **2007**, *1*, 1249-1253, doi:10.1017/S1751731107000651.

100. Yuan, J.F.; Moaeen-ud-Din, M.; Gong, Y.Z.; Peng, X.L.; Yang, L.G.; Feng, Y.P.; Liu, J.; Hu, B.; Affara, N.A.; Jafer, O.; et al. Identification of mutations of zona pellucida glycoprotein (ZP3) and its association with pig reproductive traits. *Journal of Animal Breeding and Genetics* **2007**, *124*, 144-149, doi:https://doi.org/10.1111/j.1439-0388.2007.00651.x.

101. Yurina, A.; Skovorodin, E.; Dolmatova, I. Evaluation of reproductive traits and ovarian and uterine morphology of sows with different genotypes for the estrogen receptor, prolactin receptor, and follicle-stimulating hormone subunit beta genes. *Can J Vet Res* **2021**, *85*, 186-192.

102. Zakova, E.; Wolf, J. Herd specific effects of the ESR gene on litter size and production traits in Czech Large White sows. *Czech Journal of Animal Science* **2004**, *49*, 373-382, doi:10.17221/4321-CJAS.

103. Zhang, L.; Wang, L.; Li, Y.; Li, W.; Yan, H.; Liu, X.; Zhao, K.; Wang, L. A substitution within erythropoietin receptor gene D1 domain associated with litter size in Beijing Black pig, Sus scrofa. *Animal Science Journal* **2011**, *82*, 627-632, doi:https://doi.org/10.1111/j.1740-0929.2011.00901.x.

104. Zhang, Q.; Huang, R.; Ma, X.; Jiang, N.; Zhou, W.; Gao, C.; Zhao, M.; Niu, P.; Zhang, Z.; Li, Q.; et al. Association of Rs339939442 in the AHR Gene with Litter Size are Inconsistent among Chinese Indigenous Pigs and Western Commercial Pigs. *Animals : an open access journal from MDPI* **2019**, *10*, doi:10.3390/ani10010011.

105. Zhang, W.; Fang, M.; Li, Y.; Nie, Q.; Zhang, X. Identification of TDRP1 gene and its association with pig reproduction traits. *DNA Cell Biol* **2012**, *31*, 371-377, doi:10.1089/dna.2011.1328.

106. Zhang, X.D.; Zhu, H.Y.; Zhou, J.; Wang, N.; Zhou, N.; Huang, L.; Wu, T.; Feng, Y.F.; Ding, Y.Y.; Yin, Z.J. Relationship between polymorphisms in exon 10 of FSHR gene and litter size in swine. *Genet Mol Res* **2015**, *14*, 8252-8261, doi:10.4238/2015.july.27.13.

107. Zhang, Y.; Du, H.; Chen, J.; Yang, G.; Zhang, X. Porcine growth differentiation factor 9 gene polymorphisms and their associations with litter size. *J Genet Genomics* **2008**, *35*, 163-169, doi:10.1016/S1673-8527(08)60022-3.

108. Zhang, Y.H.; Mei, S.Q.; Peng, X.W.; Niu, B.Y.; Ren, Z.Q.; Zuo, B.; Xu, D.Q.; Lei, M.G.; Zheng, R.; Jiang, S.W.; et al. Molecular characterization and SNPs analysis of the porcine Deleted in AZoospermia Like (pDAZL) gene. *Animal reproduction science* **2009**, *112*, 415-422, doi:10.1016/j.anireprosci.2008.05.069.

109. Zhang, Y.H.; Mei, S.Q.; Peng, X.W.; Zuo, B.; Lei, M.G.; Xiong, Y.Z.; Li, F.E. Molecular cloning and polymorphism of the porcine H2AFZ gene. *Animal* **2009**, *3*, 779-782, doi:10.1017/s1751731109004170.

110. Zhao, Y.; Li, N.; Xiao, L.; Cao, G.; Chen, Y.; Zhang, S.; Chen, Y.; Wu, C.; Zhang, J.; Sun, S.; et al. FSHB subunit gene is associated with major gene controlling litter size in commercial pig breeds. *Science in China Series C: Life Sciences* **1998**, *41*, 664-668, doi:10.1007/BF02882910.

111. Zhao, Z.; Liu, Y. Molecular Cloning of Porcine SUN5 Gene and Association between a SNP with Litter Size Trait. *Animal biotechnology* **2017**, *28*, 301-305, doi:10.1080/10495398.2017.1293545.

112. Zhu, H.; Yang, H.; Zhao, S.; Tian, Y.; Su, Y. Associations of genetic polymorphisms with reproduction and meat quality characteristics in Chinese Hebao pigs. *Canadian Journal of Animal Science* **2017**, *97*, 183-192, doi:10.1139/cjas-2015-0205.

113. Zhu, M.J.; Yu, M.; Liu, B.; Zhu, Z.Z.; Xiong, T.A.; Fan, B.; Xu, S.P.; Du, Y.Q.; Peng, Z.Z.; Li, K. Genetic status of ESR locus and other unidentified genes associated with litter size in Chinese indigenous Tongcheng pig breed after a long time selection. *Asian-Australas J Anim Sci* **2004**, *17*, 598-602.

114. Ziółkowska, A.; Bogdzińska, M.; Biegniewski, J. Polymorphism of prolactin receptor gene (PRLR) in the polish landrace and polish large white swine population and reproductive traits. *Journal of Central European Agriculture* **2010**, *11*, 443-448, doi:10.5513/JCEA01/11.4.859.
